# Genome-wide analysis and identification of Carotenoid Cleavage Oxygenase (*CCO*) gene family in coffee (*coffee arabica*) under abiotic stress

**DOI:** 10.1186/s12863-024-01248-4

**Published:** 2024-07-19

**Authors:** Shajiha Naeem, Yuexia Wang, Shiming Han, Muhammad Zeshan Haider, Adnan Sami, Muhammad Shafiq, Qurban Ali, Muhammad Hamza Tariq Bhatti, Arsalan Ahmad, Irfan Ali Sabir, Jihong Dong, Pravej Alam, Muhammad Aamir Manzoor

**Affiliations:** 1 School of Public Policy & Management, China University of Mining and Technology, Xuzhou, China; 2 Department of Plant Breeding and Genetics, Faculty of Agricultural Sciences, University of the Punjab, P.O BOX. 54590, Lahore, Pakistan; 3 School of Biological Sciences and Technology, Liupanshui Normal University, Liupanshui, 553004 China; 4 Department of Horticulture, Faculty of Agricultural Sciences, University of the Punjab, P.O BOX. 54590, Lahore, Pakistan; 5 College of Horticulture, South China Agricultural University, Guangzhou, 510642 China; 6 Department of Plant Science, School of Agriculture and Biology, Shanghai Jiao Tong University, Shanghai, China; 7 Department of Entomology, Faculty of Agricultural Sciences, University of the Punjab, P.O BOX. 54590, Lahore, Pakistan; 8 School of Environment and Surveying, China University of Mining and Technology, Xuzhou, Jiangsu 221116 China; 9 Department of Biology, College of Science and Humanities, Prince Sattam Bin Abdulaziz University, Alkharj, 11942 Saudi Arabia

**Keywords:** Abiotic stress, *Coffee arabica*, Drought-resistant, Carotenoids, *CCO* genes, Plant hormones

## Abstract

The coffee industry holds importance, providing livelihoods for millions of farmers globally and playing a vital role in the economies of coffee-producing countries. Environmental conditions such as drought and temperature fluctuations can adversely affect the quality and yield of coffee crops.Carotenoid cleavage oxygenases (CCO) enzymes are essential for coffee plants as they help break down carotenoids contributing to growth and stress resistance. However, knowledge about the CCO gene family in *Coffee arabica* was limited. In this study identified *21 CCO* genes in *Coffee arabica (C. arabica)* revealing two subfamilies carotenoid cleavage dioxygenases (*CCDs) and* 9-cis-epoxy carotenoid dioxygenases (*NCED*) through phylogenic analysis. These subfamilies exhibited distribution patterns in terms of gene structure, domains, and motifs. The 21 *CaCCO* genes, comprising *5 NCED and 16 CCD* genes were found across chromosomes. Promoter sequencing analysis revealed cis-elements that likely interact with plant stress-responsive, growth-related, and phytohormones, like auxin and abscisic acid. A comprehensive genome-wide comparison, between *C. arabica* and *A. thaliana* was conducted to understand the characteristics of *CCO* genes. RTqPCR data indicated that *CaNCED5, CaNCED6, CaNCED12, and CaNCED20* are target genes involved in the growth of drought coffee plants leading to increased crop yield, in a conditions, with limited water availability. This reveals the role of coffee *CCOs* in responding to abiotic stress and identifies potential genes useful for breeding stress-resistant coffee varieties.

**Supplementary Information:**

The online version contains supplementary material available at 10.1186/s12863-024-01248-4.

## Introduction

Carotenoids, the naturally occurring isoprenoids, are found in prokaryotes, fungi, bacteria, and plants [1, 2]. The carotenoid biosynthesis is carried out and implemented by the higher plants, algae, fungi, and bacteria; animals can take and consume carotenoids through the diet [3]. Furthermore, abscisic acid and strigolactone which are apocarotenoid hormones, play the role of carotenoids precursors [4]. Plants can use carotenoids for many necessary biological processes that determine plant growth and development [5]. Carotenoids are cleaved into various smaller compounds, which play crucial roles in signaling mechanisms and the production of hormones and volatile compounds [6], Carotenoids are not only the chemical precursors of phytohormones but also have very important roles in signaling transduction, growth, and development of plants [2, 7]. Many studies have already shown the various biological roles of the *Carotenoid cleavage oxygenases* (*CCO*) gene family in plants, including their implication in pigmentation, photosynthesis, protection against light, response to abiotic stresses, and the biosynthesis of aroma volatiles and plant hormones [8, 9]. Carotenoid Cleavage Oxygenases (CCOs) are the enzymes that break down the conjugated double bonds of carotenoid polyene bonds and thus form different apocarotenoids and their derivatives [10]. The *CCO*s of plants can be classified into two main subfamilies *carotenoid cleavage dioxygenase (CCD*) and *9-cis-epoxy carotenoid (NCED)* depending on whether they promote the epoxidation of their substrates [11]. As a result of this, these carotenoids, with conjunctive double bonds, are metabolized differently through *CCOs.* This process is known as carotenoid cleavage oxygenases which are a kind of dioxygenase enzyme occurring in plants [12, 13].

The 9-Cis-epoxy carotenoid dioxygenase (NCED) is a key enzyme of ABA production, which is found in almost all plants. Its subfamily shows remarkable evolutionary conservation, with little variation and significant exon preservation, emphasizing the need to maintain its structural integrity for proper functioning [14–17]. The slow evolution of *NCED* genes specifies the multiple functions in different tissues of plants [15]. The diverse expression patterns and fluctuating activities of NCED in different parts of the plant may contribute to the tolerance of the plant to drought conditions and the production of abscisic acid (ABA) hormones to prepare the plant to coordinate plant responses to environmental signals [18, 19]. The discovery of the *NCED* gene greatly advanced our understanding of ABA production and the function of this hormone in plant growth. *NCED’s* involvement first identified in the maize ABA-deletion mutant Vp14 and in its overall biosynthesis process, was elucidated [20]. Additional research was undertaken in *Arabidopsis* revealing 9 *CCO* genes, of which 5 (*AtNCED2*, *AtNCED3*, *AtNCED5*, *AtNCED6*, and *AtNCED9*) had a direct association with ABA biosynthesis. The modification of *AtNCED3* levels has been shown to boost drought tolerance through ABA accumulation [21]. *NCED* genes had the highest level of co-expression with the production of abscisic acid (ABA). All the sites of 9-cis-epoxy carotenoid cleavages of *NCED* enzymes, generated C15-xanthoxin - the potent precursor of ABA [22, 23]. The study of the *NCED* genes in several species such as Tamatim [24], cowpea [25], and rice [26] greatly increased our understanding of the evolution and functionality of NCED across plant taxa. This data put forward *NCED* as crucially vital in the ABA formation pathway that enables plants to make responses to environmental stress and developmental signals. Specifically, all *NCED* genes in plants mediate the biosynthesis of ABA and can cleave violaxanthin and neoxanthin into xanthaldehyde [27], which is the critical rate-limiting step and the first stage of ABA biosynthesis in plants [27]. After that, the xanthaldehyde is moved into the cytoplasm, where it undergoes a sequence of events that ultimately result in the formation of the plant hormone ABA. Different *NCED* genes have been studied in several plant species such as Arabidopsis [28], citrus [29], cotton [30], cucumber [31], wheat [32], and tobacco [23]. However, the *NCED* genes’ role in coffee is not completely known [12].

In the model plant *Arabidopsis thaliana*, the *CCO* family has four carotenoid cleavage dioxygenases (*CCDs) (CCD1, CCD4, CCD7, and CCD8)* [31]. This was followed by another important discovery, one of the members of the *CCO* family which was found, in *S. Lycopersicum*, and was named, *CCDL* [23, 33]. Studies of the genes of the *CCD* subfamily showed that these genes were involved in different physiological and developmental molecular processes in plants, including photosynthesis, response to abiotic or synthetic stresses, and apocarotenoids synthesis that include aromatic volatiles and strigolactones (SLs) [34, 35]. Studies show that *CCD1* and *CCD4* can selectively cleave β-carotene into the volatile, aromatic compound β-ionone. [36]. This is one of the main components of the floral scent of the plant [37]. At the same time, the cleavage activity of *CCD4* can dramatically decrease the accumulation of β-carotene in plastids, which is also the main factor that affects the color of flowers and fruits of some plants [38]. *CCD7* and *CCD8* genes in the family form a complex regulatory network in association with other genes that mediate plant development and the biosynthesis of plant hormone aurolactone in a majority of plants [39]. Gene expression can regulate *CCD7* and *CCD8* genes when plants are developing and undergoing morphogenesis [40]. *CCD* gene family has already been discovered and studied in several plant species such as Arabidopsis [41], rice [42], sorghum [42], wheat [43], watermelon [31], pepper [44], tobacco [45], rapeseed [46], and maize [23].

Genomic data for the majority of polyploids could not be obtained without requiring a significant investment of time and resources. Thus, the understanding of polyploid genome evolution has mainly been limited to model systems [47]. Recent significant advancements in DNA sequencing technologies have addressed this shortcoming. As a result, there are now exciting opportunities to study the genomes of polyploid plant species, both with and without previously sequenced genomes [48]. In this work, genetic alterations in the allopolyploid *C. Arabica* were examined using mRNA-seq data [49]. *C. arabica*, one of the most important commercial crops in tropical and subtropical developing nations, is a member of the *Rubiaceae* family, which has 124 species. It produces about 30% of the coffee beans produced worldwide [50]. *C. arabica* L. is an allotetraploid species (2n = 4x = 44) displaying a diploid-like meiotic behavior, that is believed to have been formed through the spontaneous hybridization of two diploid species, *C. canephora* and *C. eugenioides* [51]. *C. canephora* the species, also known as “robusta coffee” is considered one of the parents of *C. arabica*, which is responsible for approximately 70% of coffee production worldwide [52]. As per the latest national statistics, the average weekly consumption of coffee per person in Japan is approximately 11 cups [53]. The health effects of coffee are gaining increasing popularity [54].

This research also sheds light on the functional differentiation and evolutionary history of *CCO* genes in plants. Our primary objective was to identify the function and expression patterns of *CCO* genes in the coffee genome. We utilized RT-PCR and various bioinformatics tools to investigate their functions. The genome-wide identification and characterization conducted in this study will serve as a foundation for the cloning and functional analysis of these genes.

## Materials and methods

### Sequence retrieval

We obtained the gene annotation files of *C. Arabica* from the Phytozome database (https://phytozome-next.jgi.doe.gov/). The *A. thaliana* CCO peptide sequences were downloaded from NCBI (https://www.ncbi.nlm.nih.gov). To locate the *CCO* genes, in *C. Arabica* we utilized the RPE65 (PF03055.16) from the NCBI database as a query to perform Blast at Phytozome against the protein sequences of *C. Arabica*. The search, in the *C. Arabica* database resulted in identifying twenty-one sequences, which were cross-checked using NCBI CCD (Conserved Domain Database: http://www.ncbi.nlm.nih.gov/Structure/cdd/wrpsb.cgi) with parameters to confirm their accuracy [55].

### Physio-chemical and subcellular analysis of *CCO* gene

The ProtParam (https://web.expasy.org/protparam/) tool was used to find out details, like the protein size, molecular weight, and isoelectric point of CaCCO peptides, for gene names, chromosomal positions, and protein sequences of *CCO* proteins referred to the Phytozome. To determine where the *CCO* genes were located within organelles we relied on the WoLF PSORT (https://wolfpsort.hgc.jp/) program [56].

### Analysis of gene structure, cis-regulatory elements, and motifs

The Gene Structure Display Server (GSDS) v2.0 (http://gsds.cbi.pku.edu.cn/) was utilized to display the intron-exon structure of *CCO* genes. The PlantCare database (http://bioinformatics.psb.ugent.be/webtools/plantcare/html) was employed to analyze cis-elements (CRES) associated with these genes using 1500 upstream promoter sequences. For motifs identification the MEME suit (http://meme.nbcr.net/meme/) program, with a value of 10 motifs was utilized. The discovered motif was visualized using TB tools [57].

### Phylogenetic analysis

Phylogenetic analysis was constructed using the *CCO* amino acid (AA) sequences of *C. Arabica, A. tequilana, A. thaliana, and S. lycopersicum*. Using a bootstrapping value of 1000 replications, a phylogenetic tree was constructed from the aligned protein sequence using the maximum-joining (MJ) program in MEGA 11. ITOL (https://itol.embl.de/upload.cgi) was used to show and visualize the derived phylogenetic tree [58].

### Duplication analysis, chromosomal mapping, and synteny investigation

The divergence period of the *CCO* genes was determined using the Ka/Ks ratio, with the TB tools. Gene pairs were calculated by measuring the Ka/Ks ratio of genes [59]. The time of divergence (DT) was then calculated using the formula T = Ks/2λ, where λ represents the substitution rate (6.56*10^-9). Gene duplication events were analyzed using MCScanX v1.0 with default settings to assess collinearity. *A. thaliana* and *C. arabica* were the two crops studied for synteny and a synteny graph was created using TB tools circus module. The start and end positions of the *CCO* gene were identified in the Phytozome database and its chromosomal mapping was done with Tbtools [16].

### Protein interaction studies

The study also confirmed the protein interactions among *CaCCO* genes by utilizing the String database v0.761 (https://string-db.org). This online resource contributed to an accurate description of the complicated web of interactions among protein domains and these *CaCCO* genes of coffee [16, 56].

### Plant material and experiment design

We investigated the *C. arabica* cultivar CAPBG2008, using seeds sourced from the Department of Plant Breeding and Genetics, University of the Punjab. The seeds of CAPBG2008 were planted in controlled conditions. Drought stress was initiated at the two-leaf stage. Each cultivar had five treated plants (5T) and five controlled plants (5 C), each with three replicates. In the control group, irrigation was regularly applied, while in the drought-treated group, irrigation was withheld for two weeks. Irrigation in the control blocks was administered uniformly. After four cycles of drought stress treatments, samples were collected from this experiment [60–62].

### *CaNCED*gene expression analysis in drought-stressed leaf by qRT-PCR

RNA extraction was carried out using the RNAeasy Isolation Reagent by Vazyme, in Nanjing, China. The RNA quality was evaluated through 0.8% agarose gel electrophoresis while its purity and concentration were determined using a NanoDrop 2000 Spectrophotometer. RNA samples with OD_260_/OD_280_ ratios falling between 1.90 and 2.10 were deemed suitable for examination. To analyze the *CaNCED* gene RNA samples underwent reverse transcription into cDNA utilizing the Hifair™ II 1st Strand cDNA Synthesis SuperMix for qPCR. Subsequent qRT PCR analyses were performed on a Light Cycler 480 apparatus with a 20 µL reaction mixture was treated with 5x gDNA Eraser at 37℃ for 5 min, followed by 65℃ for 2 min. Then, 5xqRT premix II and reverse transcript enzyme mix were used. RT reaction was conducted using 37℃ for 15 min; 98℃ for 2 min, and were kept 4℃. The Livak method was employed to determine the levels of gene transcripts, with each RT qPCR analysis consisting of three replicates [62, 63] **(**Table 1**).**

**Table 1:** The primers of forward and reverse were used for qRT-PCR

Gene IDs	Forword	Reverse
*CaNCED5*	AGTGACGAGGAATTCTGCTC	CCCCGACCAAATCAACCTTT
*CaNCED6*	ATGCTTCGCTGGGTATGGGA	CATCAAGCTCCCCGTCAAAG
*CaNCED12*	CCTCCAATTCAAGAACGGCT	GTTGACAATTCTTAGCTCCG
*CaNCED13*	ACGCCCTCCAATTCCAGAAC	CTTTCTCACTTCACCGGTGA
*CaNCED20*	CCAACCCTTTATTCTCTCCG	CCGGACACAATGACTTCAAC

### Results

### Identification of CCO gene in *C.arabica*

A genome-wide analysis was conducted to identify the *CaCCO* genes within the coffee genome. The RPE65 (PF03055) domain served as the query in a BlastP search against the coffee genome hosted on Phytozome v13. Subsequently, 21 CaCCO proteins were identified and analyzed for CCO domains using the Pfam database. Furthermore, the molecular weights of *CaCCO* genes ranged between (*CaCCD11*) 51867.1 and (*CaNCED*5)18504.96 Da, averaging 53442.8581 Da. The approximate isoelectric points (pI) varied from 4.95 (*CaNCED*5) to 8.84 (*CaNCED*6), with an average of 6.182. The grand average of hydropathicity (GRAVY) ranges from − 0.808 (*CaCCD8*) to -0.167 (*CaCCD1*), with an average of -0.290. Upon closer inspection of gene orientations, it was found that twelve *CaCCO* genes were aligned in the forward direction, while the remaining ten were oriented in the reverse direction. The aliphatic index indicated a range of values from 72.96 to 90.14, with an average of 79.73, suggesting that all CaCCO proteins were likely to be stable at high temperatures (Table 2).

**Table 2:** *NCED and CCD* gene family information for 15 non-redundant genes discovered in the *C. arabica* genome

Accession number	Rename	Chromosome no.	Location		Strand	Protein length	Molecular weight	pI	GRAVY	Aliphatic index
			Start	End		(A.A)	(Da)			
*evm.model.Scaffold_465.183*	*CaCCD1*	465	2,025,817	2,027,248	forward	115	12808.63	5.3	-0.167	72.96
*evm.model.Scaffold_465.165*	*CaCCD2*	465	1,769,801	1,775,443	reverse	537	60326.81	5.57	-0.178	79.85
*evm.model.Scaffold_465.185*	*CaCCD3*	465	2,042,567	2,045,571	forward	290	32451.04	6.56	-0.377	75
*evm.model.Scaffold_465.168*	*CaCCD4*	465	1,795,348	1,800,308	reverse	604	67994.56	6.4	-0.252	78.59
*evm.model.Scaffold_624.204*	*CaNCED5*	624	2,063,680	2,064,187	forward	168	18504.96	4.95	-0.361	73.57
*evm.model.Scaffold_624.203*	*CaNCED6*	624	2,062,391	2,063,669	forward	425	46946.23	8.84	-0.224	90.14
*evm.model.Scaffold_449.93*	*CaCCD7*	449	3,382,336	3,386,951	reverse	614	67517.36	6.66	-0.196	81.78
*evm.model.Scaffold_449.94*	*CaCCD8*	449	3,408,208	3,419,982	reverse	278	31605.48	5.1	-0.808	79.53
*evm.model.Scaffold_616.20*	*CaCCD9*	616	245,882	256,820	reverse	548	61808.04	5.93	-0.263	83.72
*evm.model.Scaffold_634.1*	*CaCCD10*	634	10,721	14,343	forward	622	69880.37	5.95	-0.297	79.15
*evm.model.Scaffold_2123.6*	*CaCCD11*	2123	44,448	49,453	reverse	461	51867.1	8.02	-0.258	74.86
*evm.model.Scaffold_770.342*	*CaNCED12*	770	2,661,773	2,663,294	reverse	506	56515.47	5.71	-0.278	82.09
*evm.model.Scaffold_770.1774*	*CaNCED13*	770	12,298,501	12,300,022	forward	506	56486.34	5.56	-0.268	82.29
*evm.model.Scaffold_597.130*	*CaCCD14*	597	1,383,236	1,384,816	reverse	310	35097.94	5.49	-0.23	74.84
*evm.model.Scaffold_597.134*	*CaCCD15*	597	1,444,515	1,449,511	reverse	612	69129.9	6.82	-0.275	76.93
*evm.model.Scaffold_597.154*	*CaCCD16*	597	1,706,352	1,711,188	forward	558	62259.03	6.27	-0.336	79.52
evm.model.Scaffold_597.153	*CaCCD17*	597	1,688,712	1,694,001	forward	501	55889.42	5.92	-0.295	79.42
evm.model.Scaffold_607.19	*CaCCD18*	607	190,387	201,210	reverse	548	61788.96	5.85	-0.263	85.15
evm.model.Scaffold_263.461	*CaCCD19*	263	3,844,102	3,847,584	reverse	622	69804.36	6.04	-0.299	78.68
evm.model.Scaffold_315.871	*CaNCED20*	315	6,314,552	6,316,352	reverse	599	66209.9	6.25	-0.262	84.59
evm.model.Scaffold_2652.57	*CaCCD21*	2652	1,196,195	1,200,836	forward	613	67408.12	6.65	-0.211	81.75

**Fig. 1:**
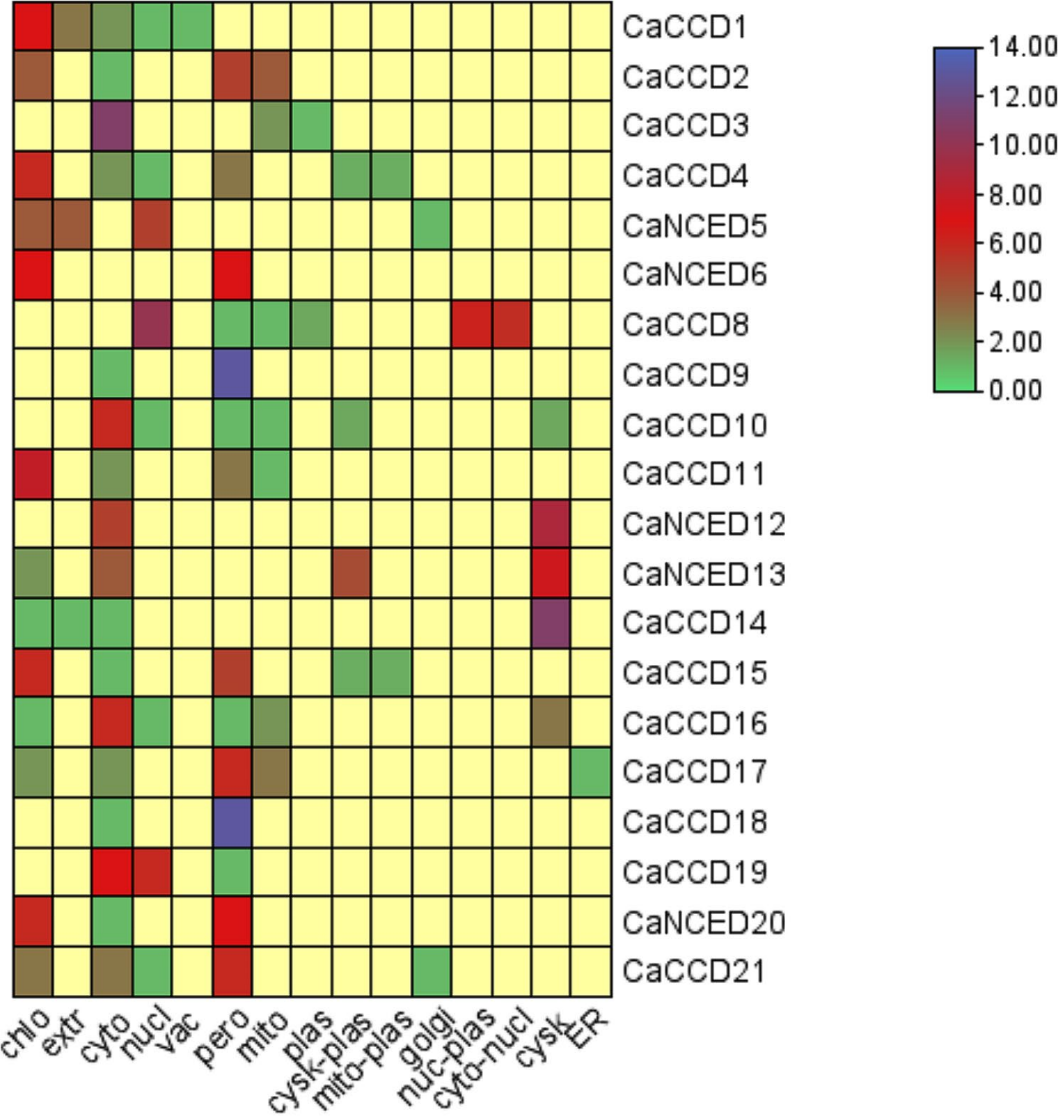
Heat map showing the subcellular locations of all *21 CaCCO* genes in the plant cell’s mitochondria, plasmid, chloroplast, nucleus, and Golgi apparatus. The color purple indicates the highest functional significance of the relevant gene in the indicated region, while the color green indicates the least functional presence of the relevant gene in the specified region

Examining the subcellular localization of the 21 *CaCCO* genes, 15 were found to be subcellularly located, with the cytoplasm, peroxisomes, and chloroplasts housing the majority of the genes. A small number were found in the nucleus and mitochondria. The smallest ones were also found, among other places, in plasmids, extracellular structures, vacuoles, and E.R (Fig. 1).

### Phylogenetic analysis

**Fig. 2:**
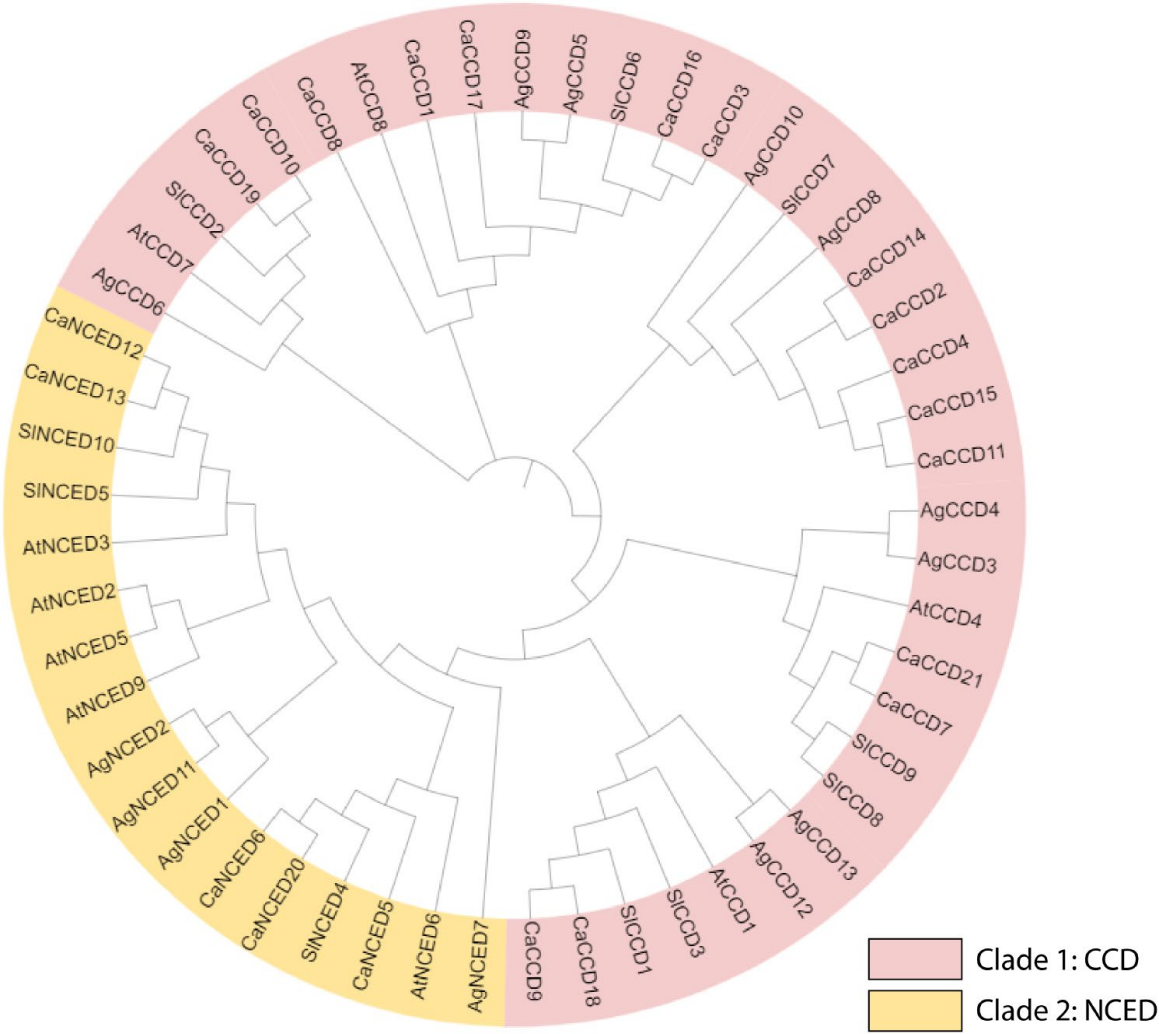
The phylogenetic relationships of *CCO* genes from four distinct crops of *C. arabica, A. thaliana, A. tequilana, and S. lycopersicum* were ascertained using a phylogenetic tree method, which is frequently used in research on Arabidopsis. The two *CCO* genes in the figure divide the four crop species according to the corresponding evolutionary features and traits. Clade I was represented by purple (*CCD*) and Clade II by orange color (*NCED*)

A phylogenetic tree was built using the reference sequences of *C. arabica (Ca), A. tequilana (Ag), Arabidopsis (At), and S. lycopersicum (Sl)* were systematically categorized into two distinct clades labeled I-II (NCED and CCD respectively). The study encompassed a total of 53 *CCO* genes, with 21 from *C. arabica* 13 from *A. tequilana*, 9 from *A. thaliana*, and 10 from *S. lycopersicum*. To enhance clarity and facilitate a comprehensive understanding of the phylogenetic relationships, each clade was denoted by a specific color scheme (Fig. 2) (S Table 1).

### Analysis of gene structure, and motifs

**Fig. 3:**
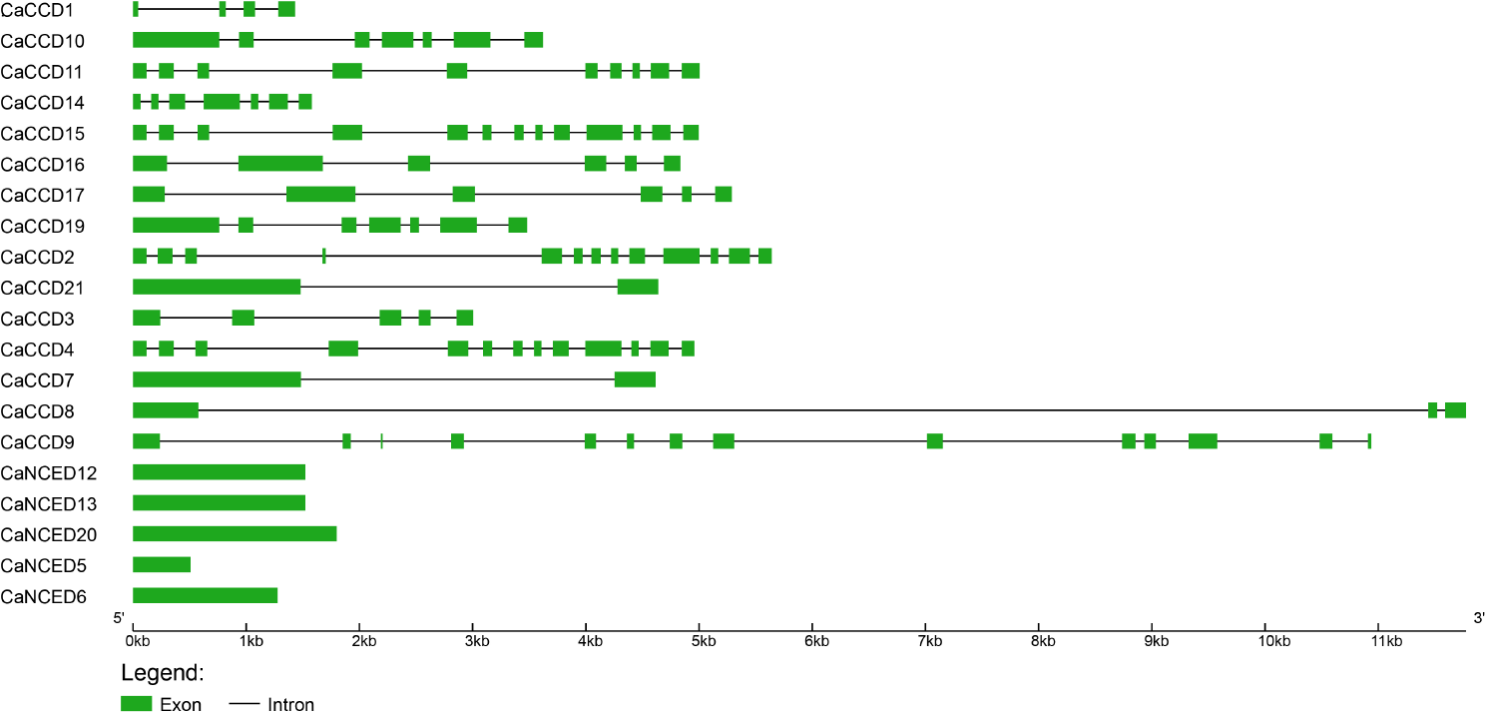
The intron-exon structure is phylogenetically represented, and it shows that *CCD* genes contain less coding sequences than *NCED* genes. The number of introns and exons in several *NCED* and *CCD* genes has remained constant

In examining the exon-intron structures of five of the twenty-one genes *CaNCED*12, *CaNCED*13, *CaNCED*20, *CaNCED*5, and *CaNCED*6 stood out with a unique profile, featuring a singular exon and an absence of introns. The three genes *CaCCD15*, *CaCCD9*, *CaCCDD2*, and CaCCD4 have thirteen exons and twelve introns. *CaCCD16* and *CaCCD17* contained six exons and five introns and *CaCCD7* had only one intron and two exons. These observations highlight significant genomic variations within the *CaCCO* gene family, highlighting the diversity in their exon-intron structure (Fig. 3**)**.

**Fig. 4:**
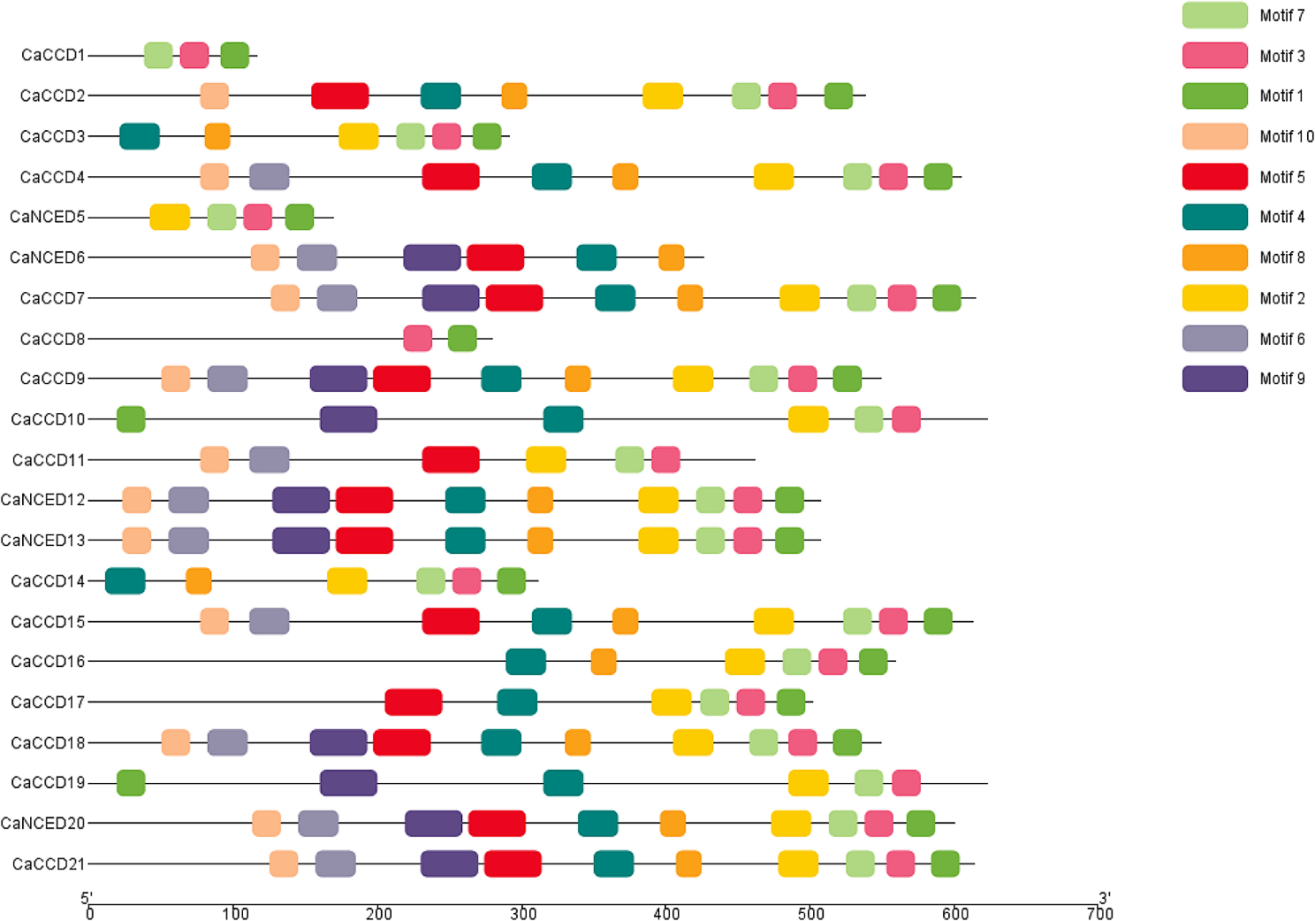
The distribution of 10 motifs along the 21 members of the *C. arabica CCO* family

Through our analysis of conserved motifs among 21 *CaCCO*, genes were subjected to motif analysis, revealing the presence of 10 different motifs. Interestingly, motifs one and seven were found to be conserved in nineteen genes. In addition, motif 3 seems to be present in all except one gene, showing that this motif plays a unique trait or regulatory function in the majority of the genes. Although, 10 motifs were found in seven genes, and six motifs were found in 8 genes, suggesting that a bunch of genes may have the same regulatory processes. This observation suggests that each *CaCCO* gene possesses distinct functions (Fig. 4).

**Fig. 5:**
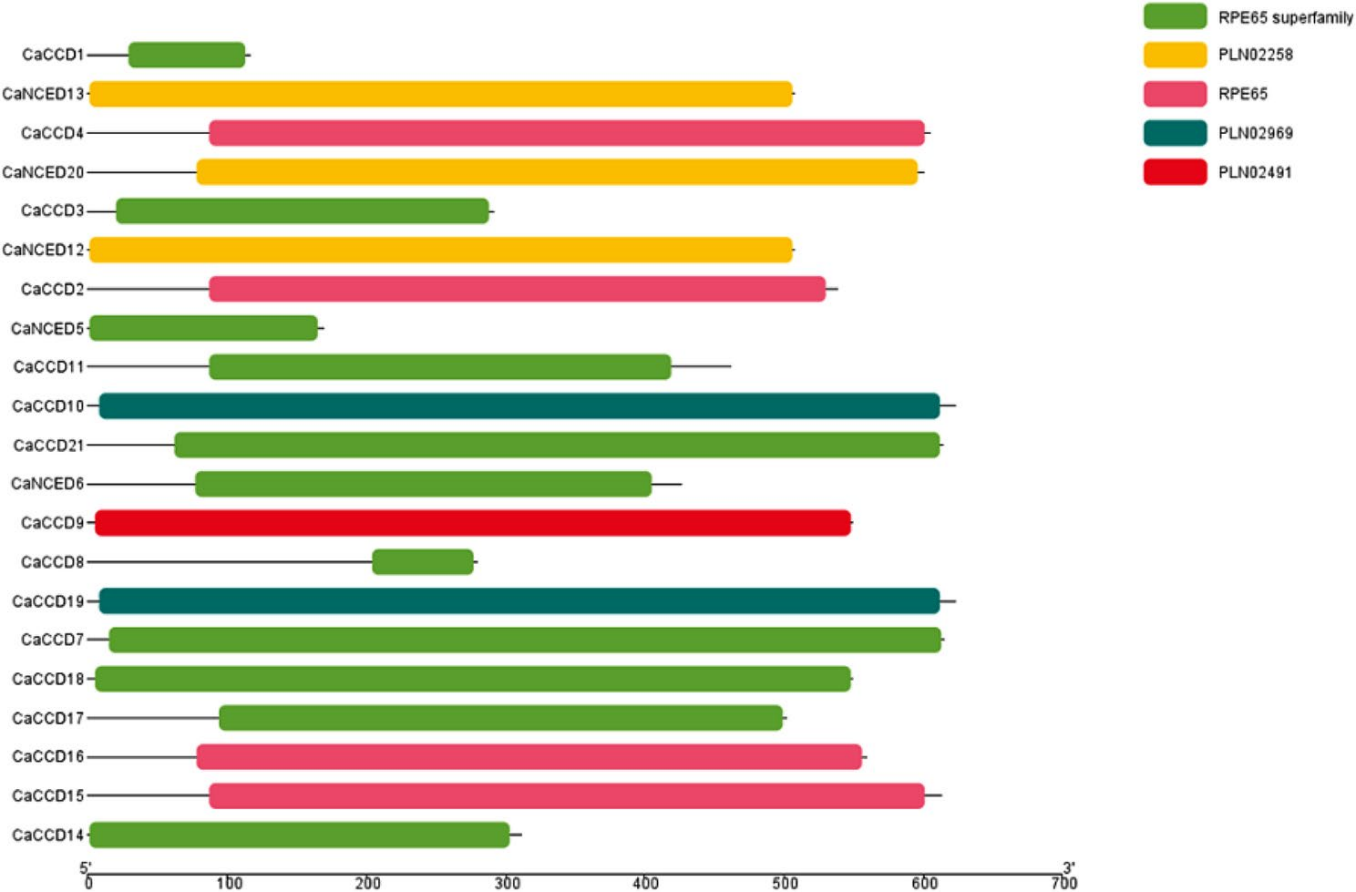
Conserved Domain analysis leads to the identification that all 21 CaCCO proteins have a conserved RPE65 superfamily domain, which demonstrates more than one particular regulatory role in plant growth and development

In the domain analysis of the 21 *CaCCO* proteins, a remarkable uniformity was observed, with all proteins featuring a singular domain identified as the RPE65 superfamily. This major domain further comprises subfamilies such as RPE65, PLN02258, PLN02969, and PLN02491. Particularly, the RPE65 superfamily is present in nine CaCCD proteins and two CaNCED proteins, while PLN02258 is found in three CaNCED proteins (CaNCED12, CaNCED13, and CaNCED20) exclusively. Moreover, RPE65 is exposed in 4 proteins of CaCCD, PLN02969 in 2 proteins of CaCCD and a single protein of CaCCD (CaCCD9). This demonstrates the conservation of the RPE65 domain in all 21 CaCCO proteins (Fig. 5).

### Evaluation of duplication event of *C. Arabica*

**Fig. 6:**
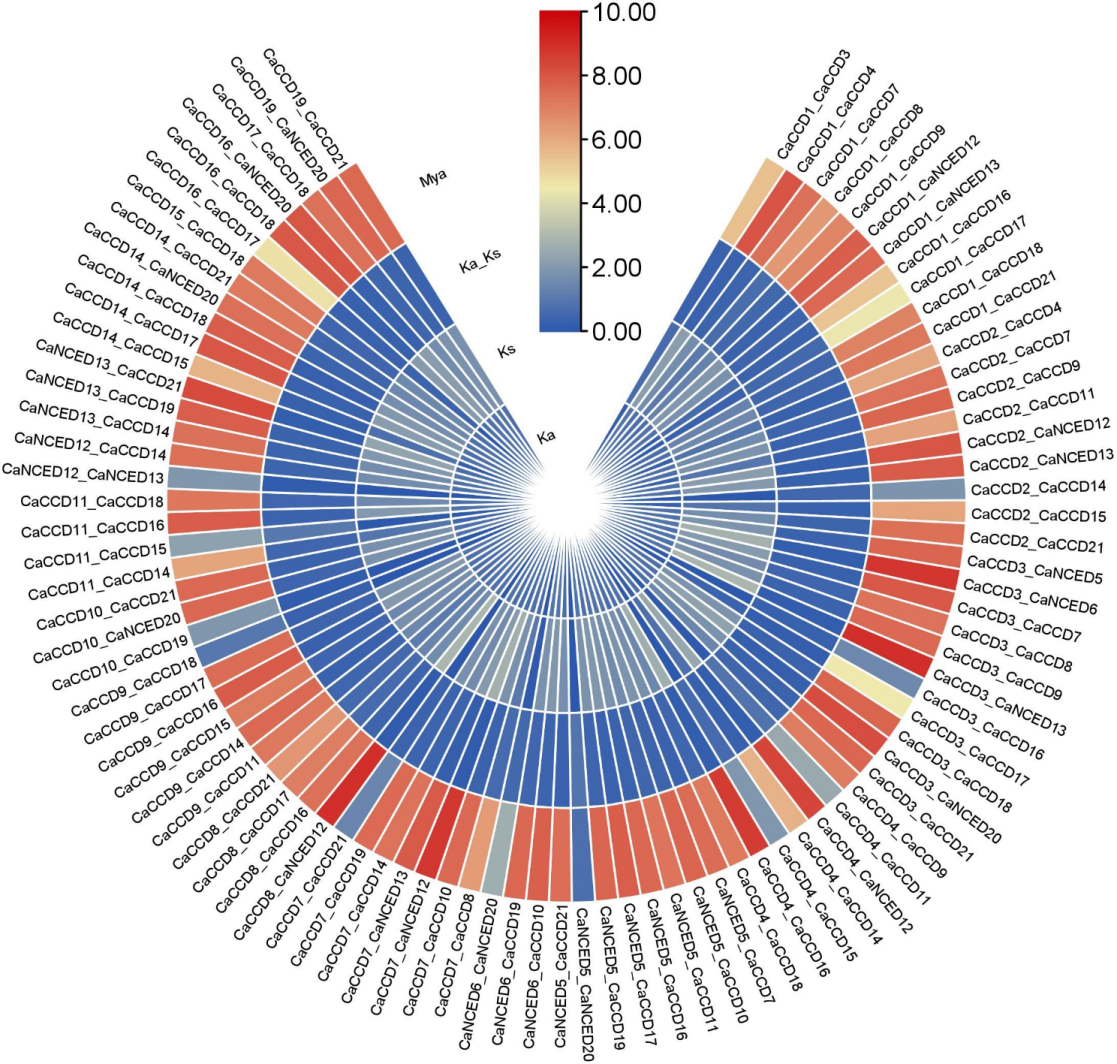
The expression ka/ks represents the ratio of mutations involving synonymous substitutions (ks) to mutations involving non-synonymous substitutions (ka). The gene duplication over selection and evolutionary pressure to paralogous pairings of *C. Arabica* genes were calculated based on ks and ka values

During the investigation of Ka/Ks ratios, there were 90 duplicated pairs of *CaCCO* genes identified in the coffee genome. The results of Ka/Ks analysis showed that *CaCCD11_CaCCD15* were found to have higher values of 1.0335. Contrariwise, CaCCD7_*CaNCED*12 showed lower Ka/Ks values (0.10843). Divergence time estimation, measured in million years ago (MYA), validates these findings. Lower MYA values for pairs like *CaNCED*5_*CaNCED*20 (0.6681) suggest more recent divergence; while higher MYA values for pairs such as CaCCD3_*CaNCED*13 (499.95) indicate ancient divergence from a common ancestor (Fig. 6)(S Table 2).

**Fig. 7:**
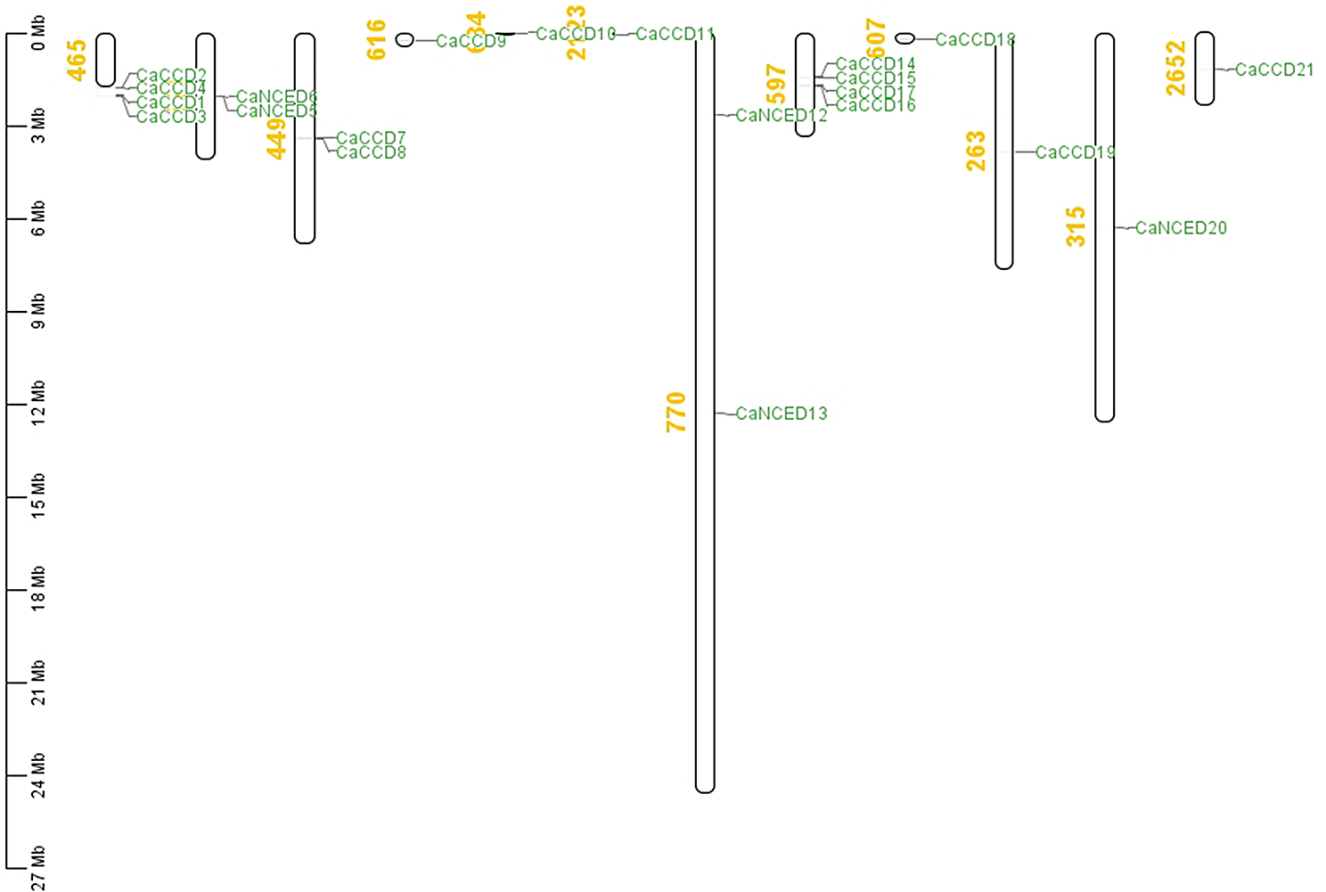
The *C. arabica* genome’s chromosomal mapping of the *CCO* genes shows the existence of paralogous copies with plausible locations. During the time of selection pressure and chromosomal rearrangement, 5 *NCED* genes suffered duplication events. Despite these alternations, the duplication of *NCED* genes remained to be able to keep their original roles and develop favorable functional traits in the *C.arabica* genome

The chromosomal localization study revealed that *CaCCO* genes were distributed across multiple scaffolds. Specifically, *CaCCD1*, *CaCCD2*, *CaCCD3*, and *CaCCD4* were located on scaffold 465, while *CaNCED*5 and *CaNCED*6 were found on scaffold 624. *CaCCD7* and *CaCCD8* were identified on Scaffold 449, *CaCCD9* on Scaffold 616, *CaCCD10* on Scaffold 634, and *CaCCD11* on Scaffold 2123. *CaNCED*12 and *CaNCED*13 were situated on scaffold 770. *CaCCD18* was present on scaffold 607, *CaCCD19* on scaffold 263, *CaNCED*20 on scaffold 315, and *CaCCD21* on scaffold 2652. Finally, *CaCCD14*, *CaCCD15*, *CaCCD16*, and *CaCCD17* were located on scaffold 597. These findings provide insights into the chromosomal organization of *CaCCO* genes (Fig. 7).

**Fig. 8:**
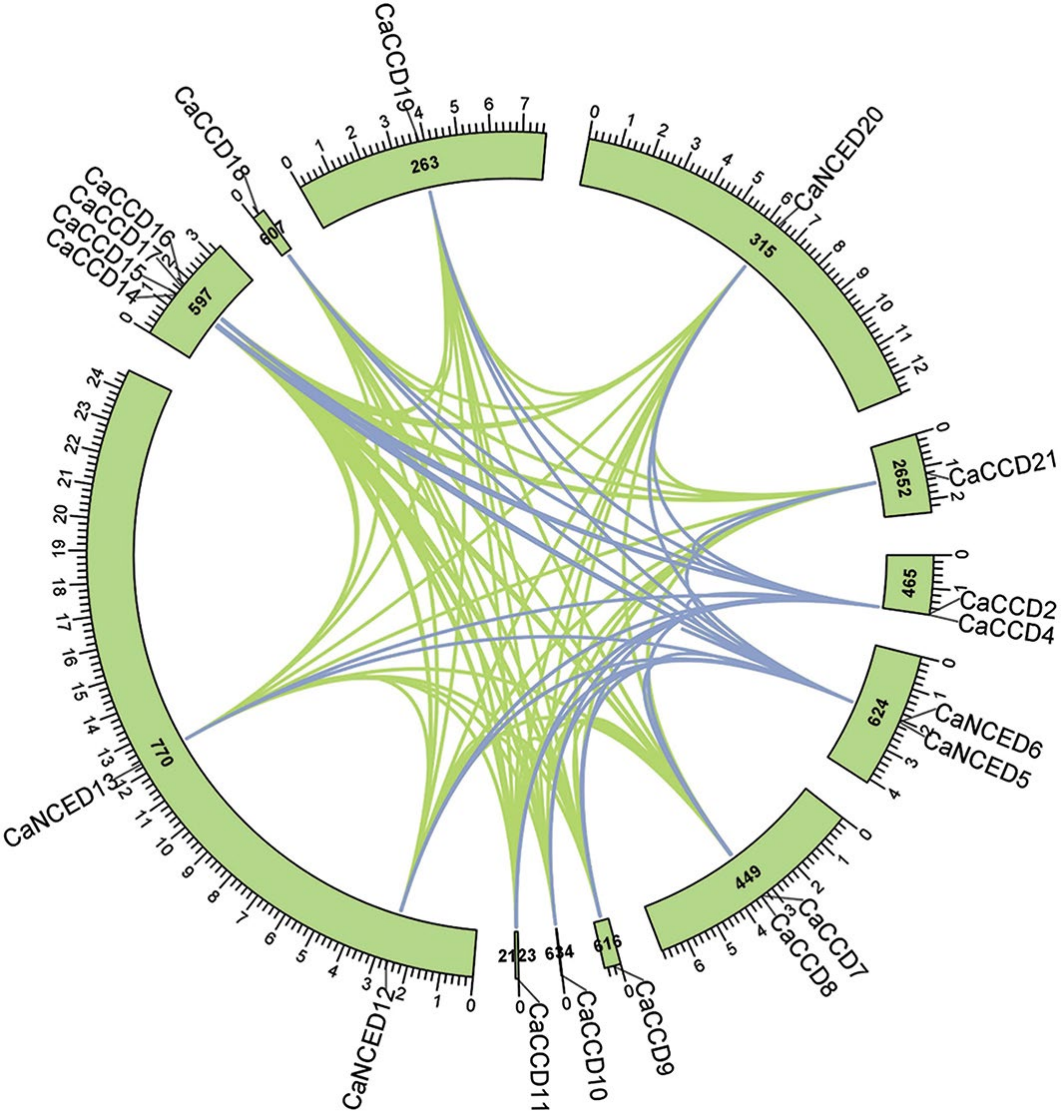
An extensive study of genome-wide synteny in *C. arabica CCO* genes revealed that genes sharing conserved areas are those with structurally similar sequences. Using blue and green hues, the method provides a visual depiction of the degree of similarity across genomic regions. A variety of dynamic processes and genomic rearrangements that enable genes to stay stable, and secure, adapt to their surroundings, and acquire new properties over time lead to gene duplication

The syntenic analysis of *CaCCO* genes reveals both segmental and tandem duplications within the *coffee* genome. Tandem duplication occurs on the same chromosome and involves the consecutive duplication of DNA segments, resulting in a repeated sequence on the same chromosome. Scaffolds 597, 449, 465 and 624, show evidence of tandem duplication. Segmental duplication can occur on the same chromosome or different chromosomes. It involves the duplication of larger genomic segments, which can be present on the same chromosome or different chromosomes while Scaffolds 770, 2652, 315,263, and 607 indicate Segmental duplication events. This comprehensive analysis highlights the paralogous duplication mechanisms shaping the coffee genome (Fig. 8).

### Analysis of *CaCCO* Cis-regulatory elements

**Fig. 9:**
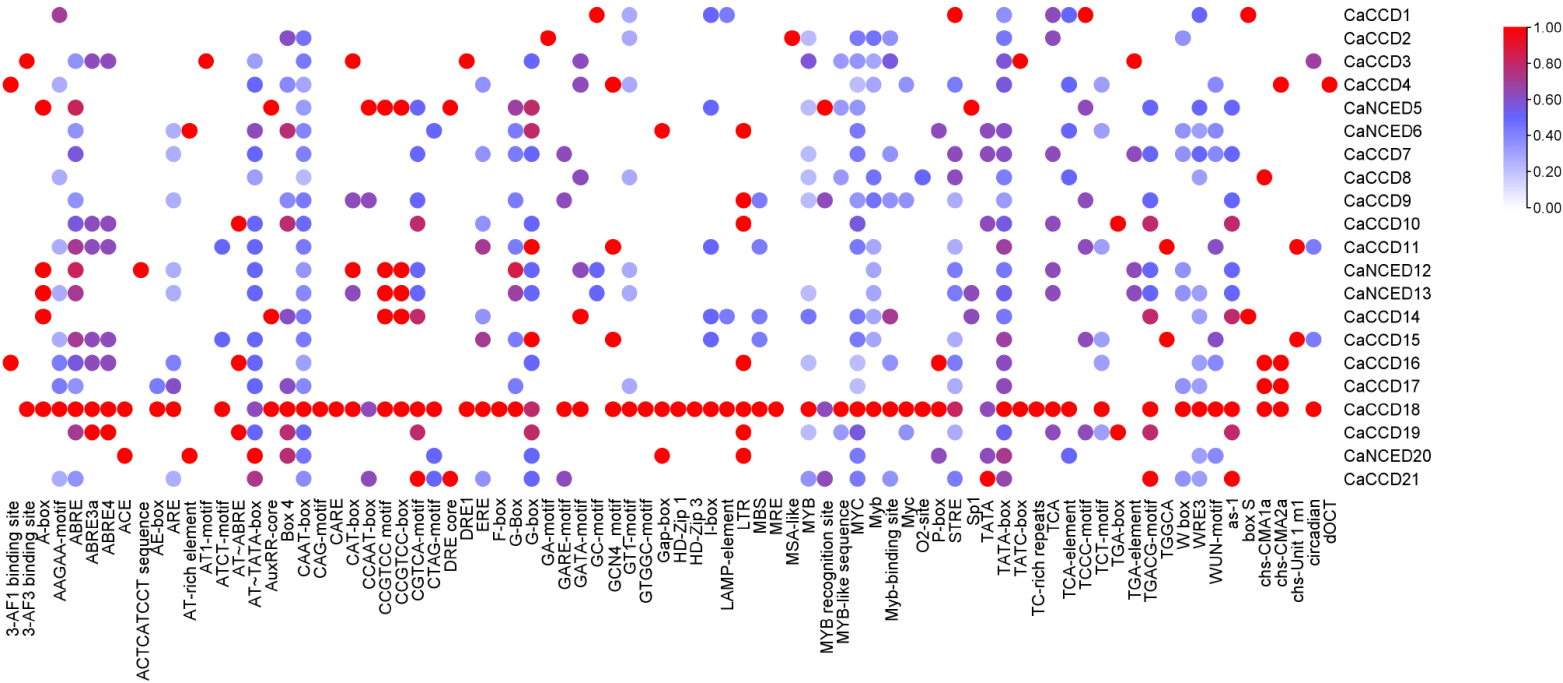
The cis-regulatory analysis of the *CCO* gene and the magnitude of each function are shown graphically. The intensity of the biochemical and physiological processes occurring in plants is ranked from red (highest) to blue (lowest)

The presence and arrangement of multiple cis-regulatory elements, in the promoter region had an impact on how genes were expressed over time. The PlantCare database was explored to investigate the roles of *CaCCO* genes in coffee plants. The results revealed a variety of cis-elements in Coffee *CCO* genes that respond to factors like light, hormones, stress, and growth. In *C. arabica* the *CCO* gene family contained 82 elements categorized into responses to phytohormones, stress-responsive, and growth-related signals. The promoter regions displayed motifs such as Skn 1_motif, GCN4_motif, MRE, Box 4, CAT box, O2 site, and circadian elements; among these were motifs like TGA element influencing auxin sensitivity and ABRE motif associated with gibberellin response. Phytohormone responses included ABRE P box TGACG motif, TCA element, and CGTCA motif linked respectively to SA (acid) ABA (acid) MeJA (methyl jasmonate), and ethylene signaling pathways. Moreover; stress-responsive elements like ARE LTR MBS W box were tied to exposure well as cold and drought stress offering valuable insights into how these regulatory mechanisms strengthened the resilience of the *CaCCO* gene family, against environmental pressures. Analysis of cis-regulatory elements revealed that a significant portion of elements (45.06%) were linked to plant hormones, containing motifs such as CGTCA-motif and TGACG-motif for MeJA response, TCA-element for SA response, GARE-motif, TATC-box, and P-box for GA response, ABRE for ABA response, and TGA-element for auxin response. The second-largest group (26%) responded to light, featuring motifs like Box 4, MRE, and G-box. The third-largest group (27%) was associated with abiotic and biotic stress, containing motifs like LTR, TC-rich repeats, ARE, MBS, and W box. (Fig. 9) (S Table 3).

### Analysis of protein-protein interaction network

**Fig. 10:**
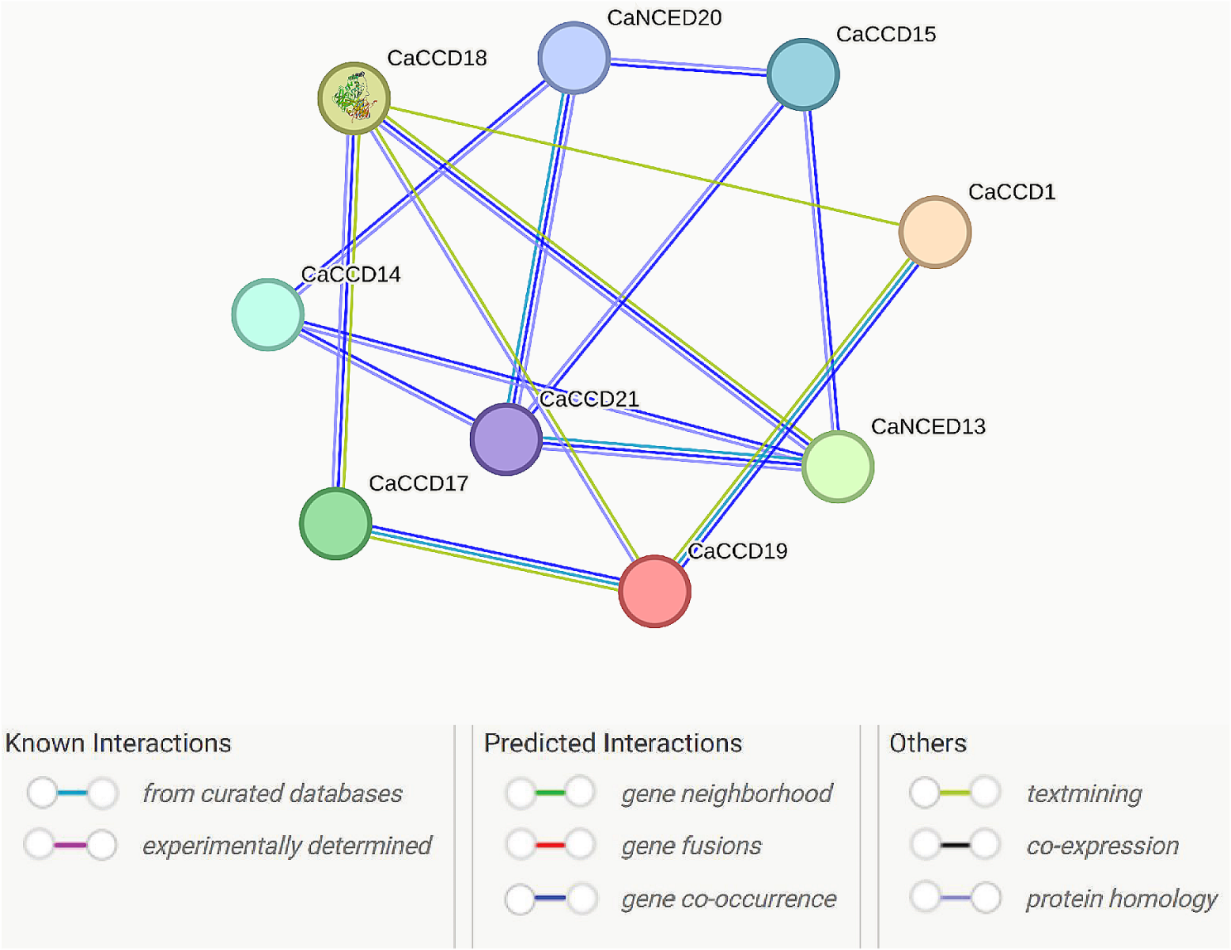
Based on protein-protein interaction, nine genes were associated with the 21 CaCCO proteins. In the dataset, CaCCD18, CaNCED13, and CaCCD21 exhibited the highest number of interactions, being associated with 9 proteins

During a protein interaction investigation, a total of 9 nodes and 14 edges were observed. The average node degree was calculated to be 14, with an average local clustering coefficient of 3.11. Interestingly, the expected number of edges in this scenario was zero, and the p-value for protein-protein interaction enrichment was remarkably low at < 1.0e-^16^, indicating a significant enrichment of interactions. To meet the minimum required interaction score, a low confidence threshold of 0.150 was applied. Among the 21 CaCCO proteins examined, interactions were observed for only nine proteins (CaCCD18, CaNCED20, CaCCD15, CaCCD1, CaNCED13, CaCCD19, CaCCD17, CaCCD21, and CaCCD14). Notably, CaCCD18, *CaNCED*13, and CaCCD21 exhibited the highest number of interactions, being associated with 9 proteins, while the remaining proteins showed associations within themselves (Fig. 10).

### Quantitative analysis of *CaNCED* gene expression in drought-stressed leaves

**Fig. 11:**
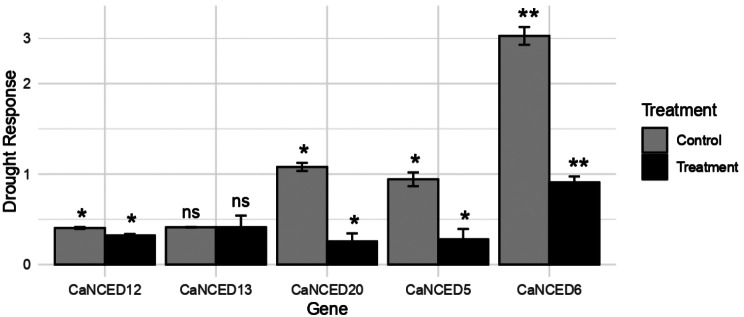
The expression of *NCED* genes in response to abiotic stress (drought stress), in controlled conditions was employed. Quantitative analysis of *CaNCED* revealed that four out of the five genes showed significant results (Less than 0.05=*, Less than 0.01=**, ns = non significant).Graph was constructed with R programming language

To clarify the expression patterns of various NCED genes in response to abiotic stress, specifically drought stress, controlled conditions were employed. Quantitative analysis of *CaNCED* revealed that four out of the five genes studied, namely *CaNCED*5, *CaNCED*6, *CaNCED*12, and *CaNCED*20, exhibited significant regulation in response to the drought conditions. Interestingly, all the significant genes displayed downregulation. Conversely, *CaNCED*13 showed upregulation under drought stress, although this change was not statistically significant (Fig. 11).

## Discussion

Drought stress severely affects coffee farming, which results in a 40–80% loss of yield and plays a role in affecting the physiology, growth, and quality of plants [60]. It makes coffee more susceptible to pests and diseases, thereby, necessitating the use of molecular techniques such as genome-wide identification of specific gene families like CCO, which are capable of combating abiotic stresses. Over the past few years, bioinformatics analysis has extensively explored the *CCO* gene family across various species [64, 65]. However, research on the *CaCCO* gene family in coffee has been comparatively limited, resulting in a knowledge gap regarding *CCO*s in coffee. This study identified and characterized 21 *CaCCO* genes in the coffee genome, shedding light on potential genes and pathways for developing resilient coffee varieties against abiotic stresses [66].

The physicochemical parameters of *CaCCO* genes in the coffee genome were evaluated to detect differences among proteins within the same clade. All identified *CCO* proteins displayed hydrophilic characteristics with negative GRAVY values, indicating an affinity for water interaction and net electrical charges at varying pH levels [31]. The aliphatic index showed that all of the 21 proteins were likely to be stable at high temperatures. Subcellular localization analysis showed divergent distribution of CaCCO proteins into chloroplasts, mitochondria, cytoplasm, cytosol, endoplasmic reticulum, nucleus, and plasma membrane. A remarkable fact was that most of the proteins were found in the Peroxisome (24%, 72 of 295), while cytoplasm and chloroplasts had equal proportions (19%, 57 of 295), implying that *CaCCO* proteins might have a significant function within these organelles [67]. Phylogenetic analysis can assist in understanding functional genomics by revealing similarities among subgroups [11, 33]. In our study, 53 *CCO* proteins with complete domain sequences were classified into two subfamilies based on sequence structures and phylogenetic relationships. The analysis identified five *CaNCED* proteins and sixteen *CaCCD* proteins, indicating potential functional similarities to *AtNCED* and *AtCCD* proteins, respectively, within this subgroup [68, 69].

Previous studies have emphasized the significance of exon-intron organization in the evolution of gene families [11]. The analysis revealed that members within the same population and clade shared similar exon, intron, and motif distributions, consistent with the structure of phylogenetic trees. While exons were universally present in all *CCO* genes, introns were absent in some proteins [70]. Notably, the *NCED* gene subfamily exhibited more conserved motifs compared to the CCD subfamily, a common feature in plant genomes. Additionally, cis-regulatory elements located within gene promoter regions play a critical role in regulating transcriptional activity and influencing gene expression patterns [70].

These findings suggest potential roles of *CaCCO* genes in regulating responses to various stresses, including drought [12]. Analyzing genomes across species offers insights into gene evolution and organization, facilitating the transfer of genomic data from well-studied taxa to less-explored ones [45]. In this study, we identified 90 pairs of paralogous genes in the genome, likely originating from gene duplication events. The duplication analysis revealed that Divergence time estimation revealed varying divergence times, with lower MYA values suggesting more recent divergence for pairs like *CaNCED*5_*CaNCED*20, while higher MYA values indicate ancient divergence for pairs such as CaCCD3_*CaNCED*13. Such duplications yield valuable insights into the expansion of gene families, a prevalent phenomenon in plants driven by tandem and segmental duplications [23].

Drought stress could reduce photosynthetic rates and transpiration in plants, resulting in crop yield losses [71]. Stomata were crucial in plant photosynthetic activities and transpiration [72]. The quantitative analysis of *CaNCED* revealed that four out of the five genes, including *CaNCED*5, *CaNCED*6, *CaNCED*12, and *CaNCED*20, exhibited significant downregulation in response to drought stress. Conversely, *CaNCED*13 showed upregulation under drought stress, although this change was not statistically significant. These findings suggested that a majority of *CaNCED* genes in coffee plants were susceptible to drought stress, as they demonstrated downregulation. These results helped build the concept of the adaptive and evolutionary value of the *CaCCO* genes. Extensive screening of a genome and detailed characterization performed by the experiment will help prepare coffee varieties resistant to abiotic stresses.

## Conclusion

In this research, twenty-one *CCO* genes were found in the coffee genome, exhibiting different intron numbers varying from one to thirteen. The existence of cis-regulatory elements that respond to light, developmental progression stages, hormone signaling, and abiotic stress within the promoter regions of the *CaCCO* gene explains their roles in coffee plants’ reaction to abiotic stress. RT-qPCR data analysis revealed that the *CaNCED5, CaNCED6, CaNCED12, and CaNCED20* genes could be useful in developing drought-resistant coffee varieties for the sake of higher yield under drought conditions. However, further research, including gene cloning and functional analysis, is necessary to confirm the significance of these genes across various physiological and biological processes.

### Electronic supplementary material

Below is the link to the electronic supplementary material.

Supplementary Material 1

## Data Availability

The data associated with this study are available in the manuscript.

## Declarations

### Data transparency

The authors ensured that all data, materials, software applications, and custom codes supported the claims made in this article and fully complied with field standards. The authors have also considered the possibility of individual journal policies regarding research data sharing, considering the norms and expectations of our discipline. Therefore, the data is available in the supplementary materials or deposited in online databases.
